# Refined obesity, smoking exposure, and lipid metrics in mortality risk assessment: a nationwide cohort analysis

**DOI:** 10.1371/journal.pone.0348128

**Published:** 2026-06-24

**Authors:** Bora Lee, Soojin Im, Sungho Won

**Affiliations:** 1 Institute of Well-Aging Medicare & Chosun University LAMP Center, Chosun University, Gwangju, Republic of Korea; 2 Institute of Health and Environment, Seoul National University, Seoul, Republic of Korea; 3 RexSoft Inc, Seoul, Republic of Korea; 4 Department of Public Health Sciences, Seoul National University, Seoul, Republic of Korea; Tehran University of Medical Sciences, IRAN, ISLAMIC REPUBLIC OF

## Abstract

**Background:**

Obesity, smoking, and lipid imbalances are well-established predictors of all-cause mortality, but conventional definitions lead to misinterpretations commonly in observational studies. This study aims to evaluate three key refinements of obesity, smoking exposure, and lipid profiles in the context of all-cause mortality using two large-scale Korean cohorts.

**Methods:**

This retrospective cohort study analyzed 659,494 participants from the Korean National Health Insurance Service-National Sample Cohort (NHIS-NSC), with external validation in 10,477 participants from the Korean National Health and Nutrition Examination Survey (KNHANES), both linked to mortality records. Obesity was classified by BMI and abdominal obesity criteria, smoking exposure was assessed using the pack-year to age ratio, and lipid abnormalities were measured using composite lipid ratios (total cholesterol/HDL≥5.0, triglyceride/HDL > 6.0, LDL/HDL≥5.0). Cox proportional hazards models were used for primary analyses, with time-dependent Cox models as sensitivity analyses.

**Results:**

In primary analyses using general Cox models, underweight individuals showed significantly elevated mortality risk across all age groups, with the combination of underweight and abdominal obesity showing a particularly high risk in those younger than 60 years (adjusted hazard ratio[AHR]=2.42, 95% confidence interval[CI]: 2.16–2.71 for underweight without abdominal obesity; AHR = 1.36, 95% CI: 0.19–9.67 for underweight with abdominal obesity). In those aged 60 years or older, being underweight without abdominal obesity was the strongest predictor (AHR = 1.79, 95% CI: 1.67–1.91). A pack-year to age ratio ≥1 was significantly associated with an increased risk of mortality (AHR = 1.65, 95% CI: 1.51–1.81). Individuals with one or more high-risk lipid profiles had an increased risk of death (AHR = 1.04, 95% CI: 1.01–1.07). Sensitivity analyses using time-dependent Cox models showed directionally consistent patterns with the primary analyses, and the findings were validated in an independent cohort (KNHANES).

**Conclusion:**

Refining obesity, smoking exposure, and lipid profile definitions led to more interpretable and clinically consistent mortality risk estimates, avoiding paradoxical findings common in observational studies. Future research would explore whether these refined metrics improve predictive accuracy compared to conventional definitions and validate their applicability in diverse populations.

## Introduction

Obesity, smoking, and dyslipidemia are well-established risk factors for all-cause mortality. However, the conventional variables used to assess these factors can introduce biases, leading to misinterpreted or counterintuitive results, especially in large-scale observational studies. For instance, relying solely on body mass index (BMI) to define obesity, categorizing smoking status only as never/former/current, or using total cholesterol (TC) alone for dyslipidemia may overlook critical nuances. Non-linear associations, unmeasured confounders, and methodological constraints such as multicollinearity further complicate these analyses, while overly broad variable definitions can impede accurate modeling and leave residual bias [1–5]. A well-known illustration is the “obesity paradox”, in which higher BMI paradoxically appears associated with lower mortality risk [6,7]. This counterintuitive finding may stem from BMI’s inability to distinguish between lean mass and fat mass, as it does not differentiate among normal-weight individuals with visceral fat, metabolically healthy obese individuals, and those with sarcopenic obesity. Similarly, simplified smoking-status categories can produce misleading conclusions; for example, some studies report higher mortality among former smokers than current smokers, potentially due to survivor bias, selective cessation in high-risk individuals, and residual effects of cumulative smoking exposure, even after cessation [8,9]. Moreover, although many studies examine multiple lipid markers such as triglycerides (TG), high-density lipoprotein cholesterol (HDL-C), and low-density lipoprotein cholesterol (LDL-C), analyzing these correlated markers simultaneously often introduces multicollinearity, complicating the interpretation of their individual effects on mortality [10,11].

A large-scale study by Ahn et al. (2017) exemplifies these methodological challenges, investigating predictors of all-cause mortality within the Korean National Health Screening Cohort (NHIS-HEALS) [6]. Although it offered valuable epidemiological insights, the study highlighted several measurement limitations. Specifically, obesity was assessed only by BMI, TC were the sole lipid measure, and smoking exposure was captured via multiple overlapping measures such as smoking status, total pack-years, smoking amount, and smoking duration – leading to potential redundancies. These observations underscore the need for more refined, integrated approaches that can better address the complex interactions among obesity, smoking and dyslipidemia when evaluating mortality risk.

In the present study, we address these limitations by introducing three key refinements in measuring obesity, smoking and dyslipidemia within a population-based cohort studies. First, rather than relying solely on BMI, we incorporates abdominal obesity to provide a clearer indication of body fat distribution and its age-related effects on mortality [12,13]. Since BMI alone cannot distinguish between lean and fat mass, this additional measures allows for a more precise estimation of adiposity-related risk. Second, instead of simple smoking status or total pack-years alone, we employ the pack-year to age ratio to quantify smoking exposure more accurately. This refined metric accounts for cumulative smoking burden relative to age and adjusts for potential survivor bias [14]. Finally, recognizing the strong interrelationship among lipid parameters, we explore composite lipid ratios (TC/HDL-c, TG/HDL-c, LDL-c/HDL-c) as alternative predictors of mortality. These ratios may serve as more robust indicators of cardiovascular and metabolic risk compared to individual lipid markers or total cholesterol alone [4,15–17].

Using a large-scale nationwide cohort with over 12 years of follow-up, we systematically examines how these enhanced metrics – abdominal obesity, the pack-year to age ratio, and composite lipid ratios – relate to all-cause mortality. To ensure the robustness and generalizability of our findings, we subsequently validate the results using an external dataset. The specific aims are to:

1. Determine how general and abdominal obesity affect mortality across different age groups
2. Assess whether the pack-year to age ratio provides a more interpretable result compared to traditional smoking metrics
3. Evaluate the role of composite lipid ratios in mortality risk, despite simultaneous inclusion of all four lipid indices
4. Validate these findings in an external dataset to ensure generalizability.

## Methods

### Data source

This study utilized data from the NHIS-National Sample Cohort (NHIS-NSC 2.2), a large-scale longitudinal retrospective cohort. As for 2022, 97.1% of Korea’s 51.4 million citizens were covered by the National Health Insurance System (NHIS), and NHIS-NSC 2.2 includes 1,137,861 individuals, which accounts for 2% of the total Korean population, sampled based on sex, age, insurance type, income level, and region [18]. NHIS-NSC 2.2 covers health records from 2002 to 2021, providing information on demographics, lifestyle factors, clinical measurements, and health examination results. For individuals with chronic diseases, the dataset includes detailed medical history, such as diagnosis dates, treatments, and disease progression.

For external validation, we used data from the Korean National Health and Nutrition Examination Survey (KNHANES), which has been conducted since 1998. KNHANES provides nationally representative data on the health and nutritional status of the Korean population, supporting public health policies [19]. This dataset includes both health examinations and self-reported surveys. Additionally, we incorporated an external validation dataset by linking KNHANES data with cause-of-death records for participants aged ≥19 years from 2007 to 2018.

To ensure privacy and confidentiality, both datasets were anonymized by the NHIS and KNHANES authorities prior to researcher access, and all the analyses were conducted within secure, restricted servers. For this study, access to the NHIS data server was granted from September 14, 2022, for a duration of six months, and to the KNHANES data server from April 15, 2024, for a period of one week.

This study was approved by the Public Institutional Review Board designated by the Ministry of Health and Welfare, Korea (Approval No.: IRB-P01-202107-21-006 for NHIS and IRB-P01-202303--01–005) and conducted in accordance with relevant ethical standards and regulations. Given the retrospective nature of the study and the use of fully anonymized secondary data from established databases (NHIS-NSC and KNHANES), the requirement for informed consent was waived by the IRB.

### Study population

Significant differences were identified in health examination items before and after 2009. Accordingly, this study focused on 700,851 individuals who underwent the NHIS Medical and Health Examination between 2009 and 2019. We excluded subjects who (1) had health examination records within one year before and after pregnancy, (2) did not have a health examination record after turning 20 years old, (3) died from accidental or unintentional injuries, congenital disorders, or perinatal conditions [20,21], or (4) had missing covariate values. After these exclusions, 659,494 individuals remained for analysis. After these exclusions, 659,494 individuals remained for analysis and were followed from baseline examination (2009–2019) until death or December 31, 2021, contributing 9.5 years of mean follow-up duration(range: 0.1–12.9 years; median(interquartile range[IQR]: 9.2 years (6.8–11.3 years)).

For external validation, we used KNHANES data linked to mortality records, which included 12,081 participants from 2010 and 2011. After applying the same exclusion criteria, 10, 477 individuals were followed until death or end of mortality linkage, contributing 7.4 years of mean follow-up duration(range: 0.1–8.0 years; median(IQR): 7.3 years (6.9–7.9 years)). The flow chart was presented in S1 Fig.

### Obesity classification

Individuals were classified based on both general obesity measured by body mass index(BMI) and abdominal obesity by waist circumference to provide a comprehensive assessment of body fat distribution and its age-dependent impact on mortality [22]. This combined classification was pre-specified based on evidence that BMI alone cannot adequately distinguish metabolic risk, as it does not differentiate between lean mass and fat mass or capture visceral adiposity [12,13].

General obesity was defined using BMI thresholds recommended by the World Health Organization(WHO) for the Asian population [23]: underweight (BMI < 18.5 kg/m^2^), normal weight (18.5 ≤ BMI < 23.0 kg/m^2^), overweight (23.0 ≤ BMI < 25.0 kg/m^2^), or obese (BMI ≥ 25.0 kg/m^2^). For detailed analysis, we further stratified obesity categories into their subcategories (25.0–27.5, 27.5–30.0, 30.0–35.0, ≥35.0 kg/m^2^) to examine dose-response relationships.

Abdominal obesity was defined as waist circumference ≥ 90 cm in men and ≥ 85 cm in women, based on Korean-specific cutoff values [24]. By cross-classifying BMI with abdominal obesity status, we created combined phenotypes that distinguish individuals with different patterns of fat distribution, thereby addressing limitations of BMI-only classifications that can produce paradoxical findings in mortality studies [6,7].

### Smoking exposure

We hypothesized that the effect of smoking exposure on mortality accumulates over an individual’s lifespan [25]. Consequently, we employed the pack-year to age ratio – a metric that quantifies cumulative smoking exposure relative to age – to reduce potential survivor bias [26]. The pack-year to age ratio was calculated as follows:


$$\begin{array}{c} Pack-year\;to\;age\;ratio\;=\;\frac{Total\;pack-years}{Current\;age\;(years)} \end{array}$$

where total pack-years = (number of cigarette packs smoked per day) x (number of years smoked).

Based on this metric, participants were classified into three categories: non-smokers (0 pack-year), low exposure (pack-year to age ratio <1.0), and high exposure (pack-year to age ratio ≥1.0). By incorporating age as a denominator, the pack-year to age ratio more accurately reflects the long-term effects of smoking relative to an individual’s lifespan, even after cessation [27,28]. A pack-year to age ratio ≥1.0 indicates that an individual has, on average, smoked at least one pack per day throughout their entire lifetime, representing particularly intensive smoking exposure. For instance, a ratio of 1.0 could correspond to smoking one pack per day for 40 years in a 40-year-old or smoking two packs per day for 30 years in a 60-year-old. This approach also helps account for the fact that the same absolute smoking burden (e.g., 30 pack-years) represents a substantially different proportion of lifetime exposure for a 40-year-old versus a 60-year-old, and may better reflect the biological impact of smoking intensity relative to age.

### Lipid profile

Due to the high correlation among individual lipid markers (TC, LDL, HDL, TG), simultaneous inclusion of these variables in statistical models can result in conflicting interpretations. Therefore, we employed composite lipid ratios provide clearer and clinically relevant cardiovascular and metabolic risk assessments.

Composite lipid ratios have been shown to be stronger predictors of cardiovascular disease and mortality than individual lipid parameters alone [17,28–29]. Specifically, we defined high-risk lipid profiles using the following established thresholds: TC/HDL≥5.0, LDL/HDL≥5.0, TG/HDL > 6.0,

A high-risk lipid profile was defined as meeting at least one of these three criteria [30]. This approach alleviates multicollinearity, a common challenge when analyzing multiple lipid markers simultaneously, thereby improving the stability and interpretability of the statistical models [11].

### Outcome

The primary outcome was all-cause mortality, which was identified by linkage with the Korean National Death Registry [31]. Mortality data were recorded using ICD-10 codes, and follow-up time was measured from the baseline health examination to death or the end of the study period (December 31, 2021).

### Covariates

Blood test parameters included fasting blood sugar (FBS), hemoglobin (Hgb), estimated glomerular filtration rate (eGFR), aspartate aminotransferase (AST), and gamma-glutamyl transpeptidase (γ-GTP). FBS levels were categorized into five groups following the American Diabetes Association guidelines: low (<50 mg/dL), healthy (55–99 mg/dL), prediabetes (100–125 mg/dL), diabetes (126–199 mg/dL), and hyperglycemic crisis (≥200 mg/dL) [32]. Hemoglobin, eGFR, AST, and γ-GTP were categorized based on clinical reference ranges [33].

Urine protein was assessed using dipstick tests, with results classified as proteinuria or albuminuria (≥2+) [34].

History of hypertension, diabetes mellitus, heart disease, stroke, and cancer was determined using self-reported data and NHIS-NSC diagnostic records, classified according to ICD-10 codes (e.g., diabetes: E10-E14, hypertension: I10-I15). Due to the low frequency of individual diseases, any history of these conditions was considered a risk factor for mortality. Alcohol consumption was categorized into non-heavy drinkers without disease, heavy drinkers without disease, and those with at least one disease or classified as heavy drinkers [35].

Physical activity was categorized into two groups: no participation vs. engaging in exercise at least once per week. This binary classification was adopted because detailed information on exercise intensity and duration varied across survey years and between NHIS-NSC and KNHANES, precluding reliable granular classification. Weekly participation in any exercise represents a meaningful threshold and ensures comparability across the entire study period (2009–2021) and between cohorts.

### Statistical analysis

Distributions of categorical variables were presented as frequency and percentage. Time-to-death for each participant was summed as person-year to compute the incidence rates (IRs) with 95% confidence intervals (CIs) [36].

Cox proportional hazard (PH) models were applied to estimate the association between risk factors and all-cause mortality [37]. Adjusted hazard ratios (AHRs) with 95% CIs were calculated after adjusting for age, sex, obesity status, history of cancer, smoking pack-year/age ratio, alcohol drinking, physical activity, blood pressure, fasting blood glucose, hemoglobin, AST, γ-GTP, eGFR, urine protein, and lipid ratios. To address the robustness of our findings and account for potential time-varying confounding, we additionally conducted time-dependent Cox PH model. Time-varying confounding occurs when covariates such as obesity status, lipid profiles, and other health indicators change during the follow-up period, potentially influencing mortality risk differently at various time points. This time-varying model provides a sensitivity analysis to validate the primary finding from the general Cox models.

The proportional hazards assumption for Cox models was not formally tested using diagnostic methods such as Schoenfeld residuals due to computational constraints with the large dataset. However, we employed time-dependent Cox models as a sensitivity analysis to assess whether covariate effects varied over time. The consistency of results between general and time-dependent models provides indirect evidence supporting the validity of the proportional hazards assumption for our primary analyses.

Statistical analyses were conducted at a two-sided significance level of 0.05, and we used the Statistical Package for SAS (version 9.4; SAS Institute Inc., Seoul, Korea), Rex (version 3.6.0.2), and R statistical software (version 4.2.3; R Foundation for Statistical Computing, Vienna, Austria) [38–40].

## Results

### Baseline characteristics

In the NHIS-NSC and KNHANES cohorts, 47.83% and 39.03% of participants, respectively, were aged 40 to <60 years, while 49.94% in NHIS-NSC and 45.65% in KNHANES were male (Table 1). Regarding general and abdominal obesity, most participants had a BMI between 18.5 and <27.5 without abdominal obesity (72.53% in NHIS-NSC; 68.03% in KNHANES), whereas a smaller proportion (10.19% in NHIS-NSC; 15.84% in KNHANES) had a BMI in the same range but with abdominal obesity.

**Table 1:** Baseline characteristics of each dataset.

Variable		NHIS-NSC	KNHANES
		(N = 659,494)	(N = 10,477)
Age at first examination (year)	< 40	213,081 (32.31%)	3,154 (30.10%)
	40 – < 60	315,458 (47.83%)	4,089 (39.03%)
	≥60	130,945 (19.86%)	3,234 (30.87%)
Male		329,335 (49.94%)	4,783 (45.65%)
General and abdominal obesity	<18.5 w/o AO	29,037 (4.40%)	421 (4.02%)
	18.5 to <27.5 w/o AO	478,303 (72.53%)	7,128 (68.03%)
	27.5 to <30.0 w/o AO	15,856 (2.40%)	123 (1.17%)
	30.0 to <35.0 w/o AO	2,056 (0.31%)	13 (0.12%)
	≥35.0 w/o AO	68 (0.01%)	0 (0.00%)
	<18.5 with AO	133 (0.02%)	0 (0.00%)
	18.5 to <27.5 with AO	67,216 (10.19%)	1660 (15.84%)
	27.5 to <30.0 with AO	39,749 (6.03%)	749 (7.15%)
	30.0 to <35.0 with AO	23,657 (3.59%)	330 (3.15%)
	≥35.0 with AO	3,419 (0.52%)	53 (0.51%)
Disease history of cancer		23,665 (3.59%)	287 (2.74%)
Smoking packyear/Age ratio	Non-smoker	405,592 (62.05%)	8,344 (79.64%)
	0<to <1	168,709 (25.81%)	2,038 (19.45%)
	≥1	85,193 (12.92%)	95 (0.91%)
Heavy alcohol drinking habit	None with no disease	262,2971 (39.87%)	5,407 (68.81%)
	Heavy drinking with no disease	194,918 (29.56%)	1,802 (17.20%)
	Heavy alcohol drinking or at least one disease	201,605 (30.57%)	3,187 (30.42%)
Physical activity		500,166 (75.84%)	10,377 (99.05%)
SBP and DBP (mmHg)	Normal	565,962 (85.82%)	8,319 (79.40%)
	Borderline	82,425 (12.50%)	2,074 (19.80%)
	HTN	11,107 (1.68%)	84 (0.80%)
FBG level (mg/dL)	<100	457,659 (69.40%)	7,574 (72.29%)
	100 to <126	159,651 (24.21%)	2,200 (21.00%)
	126 to <200	34,597 (5.25%)	604 (5.77%)
	≥200	7,587 (1.15%)	99 (0.94%)
Hgb level (g/dL)	Optimal	234,902 (35.62%)	4,134 (39.46%)
	Normal	349,294 (52.96%)	5,618 (53.62%)
	Anemia	75,298 (11.42%)	725 (6.92%)
AST (SGOT) (U/L)	Optimal	612,788 (92.92%)	10,047 (95.90%)
	Normal	23,246 (3.52%)	203 (1.94%)
	High	23,460 (3.56%)	227 (2.17%)
γ-GTP (U/L)	Optimal	559,332 (84.81%)	9,035 (86.24%)
	Normal	31,156 (4.72%)	464 (4.43%)
	High	69,006 (10.46%)	978 (9.33%)
eGFR (mL/min/1.73m2)	Normal to mild	608,669 (92.29%)	10,096 (96.36%)
	Mild to moderate damage	37,423 (5.67%)	314 (3.00%)
	Moderate to severe damage	13,402 (2.03%)	67 (0.64%)
Urine protein in the Dipstick test	<2+	653,584 (99.10%)	10,433 (99.58%)
	2+ or more	5,910 (0.90%)	44 (0.42%)
Total cholesterol / HDL ratio	Normal (<5.0)	582,581 (88.34%)	8,439 (80.55%)
	High (≥5.0)	76,913 (11.66%)	2,038 (19.45%)
LDL/HDL ratio	Normal (<5.0)	657,645 (99.72%)	10,466 (99.90%)
	High (≥5.0)	1,849 (0.28%)	11 (0.1%)
TG/HDL ratio	Normal (≤6.0)	588,133 (89.18%)	8,920 (85.14%)
	High (>6.0)	71,361 (10.82%)	1,557 (14.86%)
Cholesterol ratio	Otherwise	547,851 (83.07%)	7,882 (75.23%)
	At least one high§	111,643 (16.93%)	2,595 (24.77%)

AO, abdominal obesity; BMI, body mass index; SBP, systolic blood pressure; DBP, diastolic blood pressure; Hgb, hemoglobin; AST, aspartate aminotransferase; γ-GTP, gamma glutamyl transferase/transpeptidase; eGFR, estimated glomerular filtration rate; TC, total cholesterol; TG, triglycerides; HDL, high density lipoprotein; LDL, low density lipoprotein.

§ At least one high means one of (1) Total cholesterol/HDL ratio ≥ 5.0, (2) LDL/HDL ratio ≥ 5.0, or (3) TG/HDL ratio >6.0.

Abdominal obesity was defined the waist circumference (cm) ≥ 90 in men and ≥ 85 in women.

The prevalence of self-reported cancer history was very low (0.52% in NHIS-NSC; 0.51% in KNHANES). The proportion of individuals with a higher smoking pack-year to age ratio decreased as the ratio increased, but heavy smokers (ratio ≥1) comprised 12.92% of NHIS-NSC, which was notably higher compared to 0.91% in KNHANES.

Lipid profile abnormalities were more prevalent in KNHANES than in NHIS-NSC. The proportion of individuals with TC/HDL > 5.0 was higher in KNHANES (19.45%) compared to NHIS-NSC (11.66%), and TG/HDL > 6.0 was also more frequent in KNHANES (14.86% vs. 10.82%). Overall, the prevalence of individuals with at least one abnormal lipid ratio was higher in KNHANES (24.77%) compared to NHIS-NSC (16.93%).

Despite these differences in smoking pack-year to age ratio and lipid abnormalities, the overall distribution trends across categories remained consistent in both cohorts. This discrepancy likely reflects differences in sampling frames and survey methodologies between the two cohorts. NHIS-NSC includes all health insurance subscribers over a 12-year period (2009–2021), capturing cumulative smoking exposure across the entire adult lifespan, while KNHANES data come from a cross-sectional survey conducted in 2010–2011 with shorter exposure ascertainment windows. Additionally, KNHANES may have different response rates among heavy smokers, and the two surveys may use slightly different methods for collecting smoking history data.

### Association between obesity and all-cause mortality

Table 2 outlines the association between risk factors and all-cause mortality using general Cox models, with complete results in Supplementary Table S1. In the NHIS-NSC, underweight individuals (BMI < 18.5) showed significantly elevated mortality risk. Among those aged < 60 years without abdominal obesity, AHR was 2.42 (95% CI: 2.16–2.71) compared to normal weight. Severe obesity with abdominal obesity (BMI ≥35) also increased risk (AHR = 2.11, 95% CI: 1.61–2.76). For those age ≥ 60 years, underweight without abdominal obesity showed the highest incidence rate (IR) (56.27 per 1,000 person-years, Supplementary Table S1) and remained a strong mortality predictor (AHR = 1.79, 95% CI: 1.67–1.91). Moderately obesity showed lower mortality risk than the reference group, potentially reflecting survival bias.

**Table 2:** Risk assessment for all-cause mortality using standard Cox models.

Variable	AHR (95% CI)	
	NHIS-NSC	KNHANES
General and abdominal obesity per age		
Age < 60 / No abdominal obesity / BMI < 18.5	2.42 (2.16-2.71)***	2.82 (1.22-6.50)*
Age < 60 / No abdominal obesity / 18.5 ≤ BMI < 27.5	1 (reference)	1 (reference)
Age < 60 / No abdominal obesity / 27.5 ≤ BMI < 30.0	0.81 (0.68-0.97)*	0.71 (0.10-5.11)
Age < 60 / No abdominal obesity / 30.0 ≤ BMI < 35.0	1.35 (0.89-2.06)	NA
Age < 60 / No abdominal obesity / BMI ≥ 35.0	1.51 (0.21-10.69)	NA
Age < 60 / Abdominal obesity / BMI < 18.5	1.36 (0.19-9.67)	NA
Age < 60 / Abdominal obesity / 18.5 ≤ BMI < 27.5	1.02 (0.94-1.09)	0.67 (0.37-1.21)
Age < 60 / Abdominal obesity / 27.5 ≤ BMI < 30.0	0.89 (0.81-0.98)*	1.00 (0.48-2.08)
Age < 60 / Abdominal obesity / 30.0 ≤ BMI < 35.0	1.18 (1.05-1.33)**	0.34 (0.05-2.42)
Age < 60 / Abdominal obesity / BMI ≥ 35.0	2.11 (1.61-2.76)***	NA
Age≥60 / No abdominal obesity / BMI < 18.5	1.79 (1.67-1.91)***	1.02 (0.60-1.73)
Age≥60 / No abdominal obesity / 18.5 ≤ BMI < 27.5	0.94 (0.89-0.98)**	0.83 (0.57-1.21)
Age≥60 / No abdominal obesity / 27.5 ≤ BMI < 30.0	0.77 (0.67-0.88)***	0.75 (0.18-3.13)
Age≥60 / No abdominal obesity / 30.0 ≤ BMI < 35.0	0.96 (0.68-1.38)	NA
Age≥60 / No abdominal obesity / BMI ≥ 35.0	1.62 (0.67-3.90)	NA
Age≥60 / Abdominal obesity / BMI < 18.5	1.99 (1.37-2.89)***	NA
Age≥60 / Abdominal obesity / 18.5 ≤ BMI < 27.5	0.87 (0.83-0.92)***	0.92 (0.61-1.38)
Age≥60 / Abdominal obesity / 27.5 ≤ BMI < 30.0	0.79 (0.74-0.85)***	0.61 (0.36-1.03)
Age≥60 / Abdominal obesity / 30.0 ≤ BMI < 35.0	0.80 (0.73-0.88)***	0.45 (0.23-0.88)*
Age≥60 / Abdominal obesity / BMI ≥ 35.0	1.10 (0.84-1.44)	0.31 (0.04-2.31)
**Smoking packyear/Age ratio**		
Non-smoker	1 (reference)	1 (reference)
0<to <1	1.36 (1.32-1.40)***	0.87 (0.71-1.06)
≥1	1.65 (1.51-1.81)***	1.31 (0.79-2.17)
**Cholesterol ratio**		
Otherwise	1 (reference)	1 (reference)
At least one high§	1.04 (1.01-1.07)*	1.10 (0.92-1.32)

NA, not available(not estimable due to insufficient number of events in this subgroup); IR, incidence rate; AHR, adjusted hazard ratio; CI, confidence interval;

BMI, body mass index; TC, total cholesterol; TG, triglycerides; HDL, high density lipoprotein; LDL, low density lipoprotein.

*, p < 0.05; **, p < 0.01; ***, p < 0.001

Abdominal obesity was defined the waist circumference (cm) ≥ 90 in men and ≥ 85 in women.

§ At least one high means one of (1) TC/HDL ratio ≥ 5.0, (2) LDL/HDL ratio ≥ 5.0, or (3) TG/HDL ratio >6.0.

AHR was computed from adjustment for age, sex, obesity, history of cancer, smoking packyear/age, alcohol drinking, physical activity,

SBP and DBP, FBG level, hemoglobin, AST, γ-GTP, eGFR, urine protein, and cholesterol ratio.

Results are from standard Cox proportional hazards models. Time-dependent Cox model results are presented in Supplementary S1 Table.

Estimates for subgroups with very small sample sizes should be interpreted with caution due to wide confidence intervals reflecting limited observations.

These findings were reflected in the KNHANES validation with consistent patterns despite smaller sample size. Time-dependent models generally confirmed the results from the general Cox models (Supplementary Table S1). While the time-dependent model suggested a stronger association for younger underweight individuals with abdominal obesity (AHR = 6.43, 95% CI: 2.41–17.17), this estimate should be interpreted with caution given the small sample size in this subgroup and the wide confidence interval observed in the primary Cox model (AHR = 1.36, 95% CI: 0.19–9.67).

### Association between smoking exposure and mortality

A cumulative dose-dependent relationship was observed between cumulative smoking exposure and all-cause mortality (Table 2). In NHIS-NSC, compared to non-smokers, individuals with low exposure(a pack-year to age ratio <1) exhibited a significantly higher mortality risk (AHR = 1.36, 95% CI: 1.32–1.40), while those with high exposure(a pack-year to age ratio ≥1) demonstrated the greatest risk (AHR = 1.65, 95% CI: 1.51–1.81). The result from KNHANES showed consistent direction though not statistically significant for high exposure due to smaller sample size. Time-dependent models confirmed the dose-dependent relationship: AHR = 1.31 (95% CI: 1.27–1.35) for low exposure and AHR = 1.75 (95% CI: 1.59–1.93) for high exposure (S1 Table).

### Association between lipid profile and mortality

Participants with at least one high-risk lipid ratio (TC/HDL ≥ 5.0, LDL/HDL ≥ 5.0, TG/HDL > 6.0) had an elevated risk of mortality in NHIS-NSC(AHR = 1.04, 95% CI: 1.01–1.07; Table 2). KNHANES showed similar estimates (AHR = 1.10, 95% CI: 0.92–1.32) though non-significant due to smaller sample size. Time-dependent models showed stronger association (AHR = 1.15, 95% CI: 1.11–1.18; S1 Table), suggesting composite lipid ratios provide meaningful prognostic value beyond individual markers.

### Reassessment across age group

To investigate how mortality risk factors vary over different life stages, we categorized participants into three age groups: younger than 40 years, 40–59 years, and 60 years or older. Then, the three key refinements were reassessed in mortality risk (Table 3).

**Table 3:** Adjusted hazard ratio with 95% CI per age group.

Variable	AHR (95% CI) from Cox PH regression model in NHIS-NSC		
	Age < 40	40≤ Age < 60	Age ≥ 60
General and abdominal obesity			
BMI < 18.5 without AO	1.28 (1.01-1.62)*	2.22 (2.04-2.41)***	1.98 (1.91-2.04)***
18.5≤BMI < 30.0 without AO	1 (reference)	1 (reference)	1 (reference)
30.0≤BMI < 35.0 without AO	1.62 (0.91-2.87)	0.99 (0.70-1.40)	0.86 (0.66-1.13)
BMI≥35.0 without AO	7.51 (1.06-5.35)*	NA	1.60 (0.80-3.20)
BMI < 18.5 with AO	NA	3.70 (1.54-8.92)**	2.41 (1.88-3.09)***
18.5≤BMI < 30.0 with AO	1.00 (0.86-1.17)	0.98 (0.94-1.03)	0.91 (0.89-0.93)***
30.0≤BMI < 35.0 with AO	1.44 (1.18-1.74)***	1.02 (0.94-1.11)	0.89 (0.84-0.94)***
BMI≥35.0 with AO	2.21 (1.59-3.09)***	1.46 (1.18-1.81)***	1.38 (1.17-1.63)***
Smoking packyear/Age ratio			
Non-smoker	1 (reference)	1 (reference)	1 (reference)
0<to <1	1.35 (1.19-1.54)***	1.48 (1.41-1.55)***	1.33 (1.30-1.36)***
1+	2.26 (1.12-4.58)*	1.76 (1.56-1.98)***	1.69 (1.57-1.82)***
Cholesterol ratio			
Otherwise	1 (reference)	1 (reference)	1 (reference)
At least one high§	0.86 (0.75-0.99)*	1.03 (0.98-1.07)	1.08 (1.05-1.10)***

NA, not available; AHR, adjusted hazard ratio; CI, confidence interval; BMI, body mass index; TC, total cholesterol; TG, triglycerides; HDL, high density lipoprotein; LDL, low density lipoprotein.

*, p < 0.05; **, p < 0.01; ***, p < 0.001

§ At least one hight means one of (1) TC/HDL ratio > 5.0, (2) LDL/HDL ratio > 5.0, or (3) TG/HDL ratio >6.0.

AHR was computed from adjustment for age, sex, obesity, history of cancer, smoking packyear/age, alcohol drinking, physical activity, SBP and DBP, FBG level, hemoglobin, AST, γ-GTP, eGFR, urine protein, and cholesterol ratio

Individuals classified as underweight (BMI < 18.5) had an elevated mortality risk across all age groups, with the highest risk observed in those aged 40–59 years (AHR = 2.22, 95% CI: 2.04–2.41), followed by individuals aged ≥60 years (AHR = 1.98, 95% CI: 1.91–2.04) and those younger than 40 years (AHR = 1.28, 95% CI: 1.01–1.62). The relative risk was higher in older adults, though still elevated in younger individuals. However, after age 40, the excess mortality risk associated with underweight status showed a gradual decline compared to individuals with normal weight. Abdominal obesity was associated with increased mortality risk across all age groups, with a greater impact in individuals under 40 years.

Smoking exposure demonstrated a dose-dependent association with mortality risk. Compared to non-smokers, the highest mortality risk was observed in heavy smokers (pack-year to age ratio ≥1), with AHRs of 2.26 (age < 40), 1.76 (40–59 years), and 1.69 (≥60 years). Notably, younger heavy smokers (<40 years) exhibited the largest relative risk increase (AHR = 2.26, 95% CI: 1.12–4.58).

For lipid profiles, individuals with at least one high-risk lipid ratio had an increased risk of mortality in older adults (AHR = 1.08, 95% CI: 1.05–1.10). Interestingly, among younger individuals (<40 years), a high lipid ratio was associated with a slightly lower mortality risk (AHR = 0.86, 95% CI: 0.75–0.99), which may reflect residual confounding, metabolic compensation, or survival bias.

Despite slight differences in specific risk estimates across age groups, the overall trends in obesity, smoking exposure, and lipid profile effects on mortality remained consistent.

## Discussion

This study examined the association between obesity, smoking exposure, and lipid abnormalities with all-cause mortality using refined definitions of these risk factors. Unlike conventional approaches that rely solely on BMI, total pack-years, and individual lipid markers, this study incorporated a combined classification of general and abdominal obesity, the pack-year to age ratio for smoking exposure, and composite lipid ratios [25,41,42]. These modifications were intended to make the results more clinically interpretable and to minimizes misleading findings common in observational studies.

Obesity’s effect on mortality varied by age and adiposity distribution. Among individuals younger than 60 years, underweight without abdominal obesity was a strong predictor of mortality (AHR = 2.42, 95% CI: 2.16–2.71 in primary Cox models; Table 2). Although the time-dependent Cox model suggested a notably elevated risk for underweight individuals with abdominal obesity in this age group (AHR = 6.43, 95% CI: 2.41–17.17; Supplementary Table S1), the corresponding estimate from the primary model was underpowered due to the small sample size (AHR = 1.36, 95% CI: 0.19–9.67; Table 2). Among individuals aged 60 and older, underweight without abdominal obesity remained the strongest mortality predictor (AHR = 1.79, 95% CI: 1.67–1.91; Table 2). These findings suggest that underweight status is a consistent and strong predictor of mortality across age groups, and that the role of abdominal obesity may differ by age, although estimates for rare phenotypes such as underweight with abdominal obesity require further investigation with larger samples [43,44].

By cross-classifying BMI with abdominal obesity status, our approach distinguished several clinically important adiposity phenotypes: (1) sarcopenic obesity (underweight with abdominal obesity), characterized by loss of muscle mass with visceral fat accumulation and associated with the highest mortality risk in younger adults; (2) metabolically unhealthy normal weight (normal BMI with abdominal obesity), reflecting central adiposity despite normal body weight; (3) metabolically healthy obesity (elevated BMI without abdominal obesity), representing obesity without excess visceral fat; and (4) various gradations of obesity with central adiposity. This refined classification helps explain the ’obesity paradox’ by revealing that not all individuals within the same BMI category have equivalent metabolic risk, and that visceral adiposity–rather than total body weight alone–is a critical determinant of mortality risk. Additionally, the combined effects of general and abdominal obesity on mortality differed across age groups, with a stronger association observed in younger individuals. These findings highlight the important of considering both BMI and abdominal obesity simultaneously, particularly in age-stratified analyses.

For smoking exposure, the pack-year to age ratio was introduced to allow for a more detailed assessment of cumulative smoking burden. The results confirmed a dose-dependent relationship between smoking exposure and mortality, with heavy smokers (pack-year to age ratio ≥ 1) consistently having the highest mortality risk across age group (AHR = 2.26, 95% CI: 1.12–4.58 for age < 40; AHR = 1.76, 95% CI: 1.56–1.98 for 40–59 years; AHR = 1.69, 95% CI: 1.57–1.82 for ≥60 years). Notably, the strongest relative risk was observed in young smokers, which strengthens the long-term consequences of early smoking exposure [45,46]. Unlike total pack-years, which does not take into account differences in smoking duration relative to age [47], the pack-year to age ratio provided results that were easier to interpret and more consistent with established clinical knowledge on smoking risk.

For lipid profiles, this study examined composite lipid ratios to provide clinically meaningful interpretation while minimizing interpretative conflicts due to opposing risk implications among correlated lipid markers. Individuals with at least one high-risk lipid ratio (TC/HDL≥5.0, LDL/HDL≥5.0, TG/HDL > 6.0) showed an increased risk of mortality in primary analyses (AHR = 1.04, 95% CI: 1.01–1.07), particularly pronounced in older adults (AHR = 1.04, 95% CI: 1.05–1.10). Composite lipid ratios (TC/HDL, LDL/HDL, TG/HDL) offer practical advantages over individual lipid markers by reducing interpretative conflicts that may arise due to opposing risk implications of correlated lipid components. Several prior studies support the superior predictive power of these ratios for cardiovascular and metabolic diseases [4,15,17]. Therefore, the use of lipid ratios is not merely a statistical approach but a clinically driven decision aimed at enhancing interpretability and validity in risk assessment models.

Among individuals younger than 40 years, those with high-risk lipid ratios showed paradoxically lower mortality risk (AHR = 0.86, 95% CI: 0.75–0.99). This counterintuitive finding may reflect several factors. First, residual confounding from unmeasured lipid-lowering treatments or lifestyle interventions initiated after baseline may have reduced mortality risk in this group. Second, survival bias may have selected individuals with protective genetic factors. Third, the latency period for lipid-related cardiovascular mortality typically spans decades, and our follow-up may have been insufficient to observe the full impact of dyslipidemia in younger adults. Finally, metabolic compensation mechanisms may be more robust in younger individuals. Importantly, this paradoxical association was limited to those under 40 years and did not persist in older age groups, where high-risk lipid ratios showed expected positive association with mortality. This age-dependent pattern suggests the inverse association in younger adults reflects methodological limitations or biological latency rather than a true protective effect.

While this study demonstrated that refined metrics produce more interpretable and clinically consistent mortality risk estimates, we did not formally compare model performance (e.g., c-statistics, Akaike Information Criterion) between refined and conventional definitions. Such comparisons would require a different study design specifically aimed at predictive model development, whereas our focus was on improving the validity and interpretability of epidemiological associations. The refined metrics were evaluated based on: (1) consistency across general and time-dependent Cox models, (2) validation in an independent cohort, and (3) alignment with established clinical knowledge. Future studies could build upon these findings to formally compare predictive performance and develop clinical risk prediction tools.

Several limitations should be acknowledged. First, formal diagnostic testing of the proportional hazards assumption using Schoenfeld residuals was not performed due to computational constraints associated with the large dataset (N = 659,494). This remains as a methodological limitation. However, time-dependent Cox models were employed as a complementary approach, and the consistency of results between the primary and time-dependent models provides indirect evidence supporting the proportional hazards assumption. Further studies should incorporate the conventional diagnostics, potentially using subsampling or stratified approaches to manage computational burden. Second, this study was limited to Korean populations. The generalizability of our refined metrics to other ethnic groups with different body composition patterns, smoking behaviors, and lipid profiles remains to be established. External validation in diverse populations is needed. Third, our analysis focused on all-cause mortality and did not examine cause-specific mortality. Fourth, we were unable to incorporate information on lipid-lowering medication use or changes in treatment during follow-up, which may have influencing the observed associations. Fifth, despite adjusting for multiple confounders, residuals confounding from unmeasured factors (e.g., dietary patterns, genetic factors, socioeconomic variables) cannot be entirely excluded in observational studies. Additionally, history of chronic diseases including cancer, heart disease, stroke, hypertension, and diabetes was combined into a single binary variable due to low individual frequencies. This pragmatic approach may mask important differences in mortality risk profiles among these conditions, as the prognostic implications of a cancer history likely differ from those of hypertension alone. Future studies with larger sample sizes should consider modeling these conditions separately.

Across all analyses, refinement of obesity, smoking exposure, and lipid abnormalities led to mortality risk estimates that were more aligned with clinical expectations and less prone to the paradoxical findings commonly reported in observational studies. For instance, in contrast to studies where obese individuals appeared to have lower mortality risk than normal-weight individuals, incorporating abdominal obesity in the classification helped reduce these inconsistencies. Similarly, while some previous studies have reported unexpectedly lower mortality rates among smokers, the use of the pack-year to age ratio allowed for a more clinically plausible gradient of risk, showing a consistent relationship between cumulative smoking burden and increased mortality risk.

## Conclusion

This study demonstrated that refining obesity, smoking exposure, and lipid profile measurements led to more clinically interpretable mortality risk estimates. Combining BMI with abdominal obesity distinguished metabolically distinct phenotypes and eliminated paradoxical findings. The pack-year to age ratio provided clearer smoking dose-response relationships. Composite lipid ratios enabled evaluation of multiple correlated markers without multicollinearity issues.

Although this study did not compare predictive performance with conventional definition directly, the refined metrics were validated through modeling consistency, independent cohort replication, and alignment with clinical knowledge. These findings demonstrate that carefully refined risk factor definitions can improve the interpretability and validity of epidemiological research, addressing longstanding paradoxes in observational mortality studies.

## Supporting information

S1 Fig Flow chart of data process. (TIF)

S1 Table Risk assessment for mortality. Time-dependent Cox model results referenced in the main text.
(PDF)
