# Supplementary figures and images for: Refined obesity, smoking exposure, and lipid metrics in mortality risk assessment: a nationwide cohort analysis
Source: PLoS One. 2026 Jun 24;21(6):e0348128. doi: 10.1371/journal.pone.0348128 (PMC13293439; doi:10.1371/journal.pone.0348128)

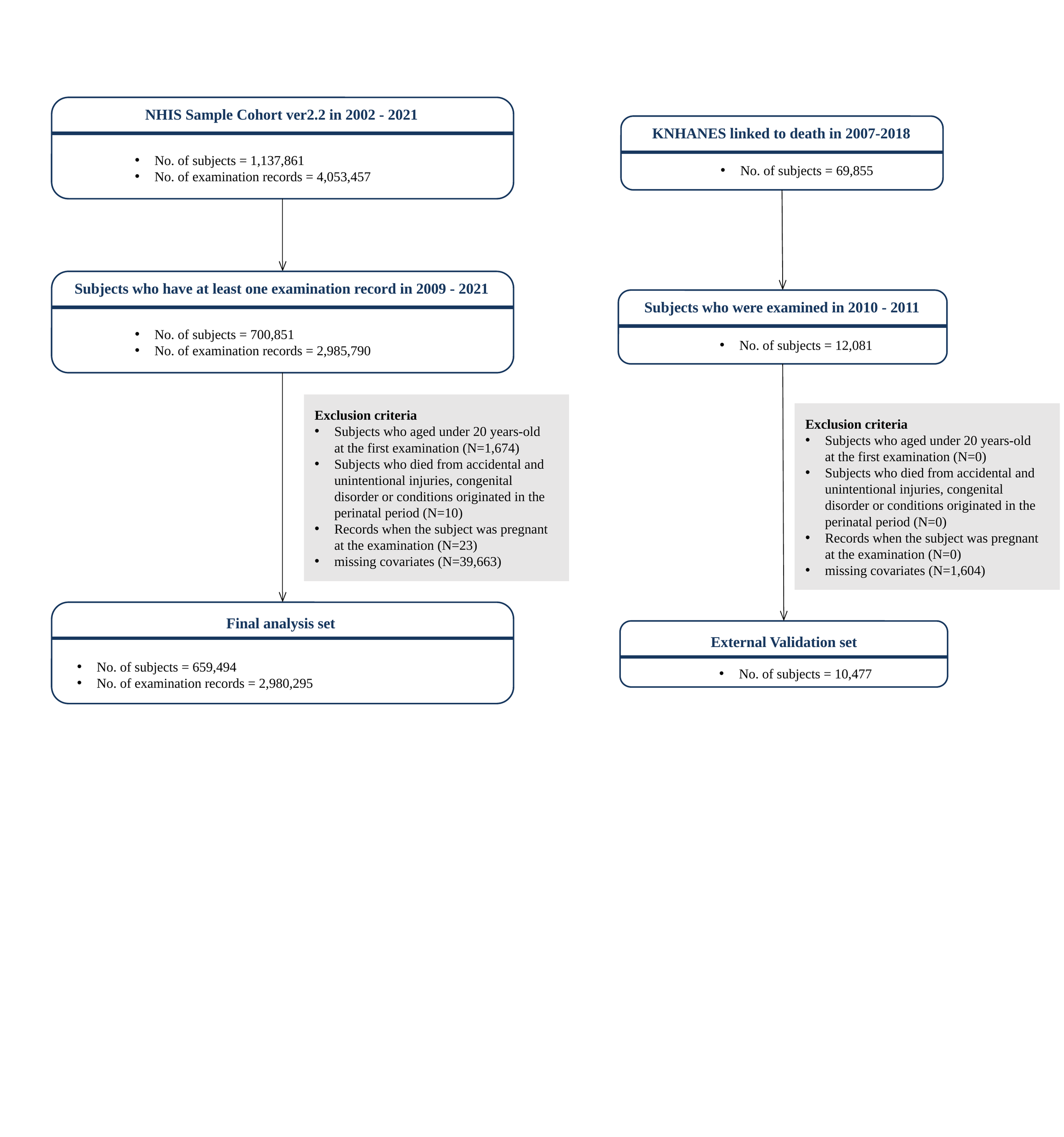

Supplement: S1 Fig — (TIF) [file pone.0348128.s001.tif]
