# Supplementary material for: Refined obesity, smoking exposure, and lipid metrics in mortality risk assessment: a nationwide cohort analysis
Source: PLoS One. 2026 Jun 24;21(6):e0348128. doi: 10.1371/journal.pone.0348128 (PMC13293439; doi:10.1371/journal.pone.0348128)
Supplement: S1 Table — Time-dependent Cox model results referenced in the main text. (PDF) [file pone.0348128.s002.pdf]

Supplementary Table 1. Risk assessment for mortality

Variable	IR (95% CI) (per 1,000 person-years)	Adjusted HR (95% CI)		Time-varying adjusted HR (95% CI)
		NHIS-NSC	KNHANES	NHIS-NSC
**Age (year)**	-	1.121 (1.119-1.122)***	1.12 (1.10-1.13)***	1.108 (1.106-1.109)***
**Sex**				
Male	5.767 (5.683, 5.852)	1.768 (1.716-1.821)***	2.07 (1.65-2.61)***	1.767 (1.716-1.820)***
Female	3.885 (3.816, 3.955)	1	1	1
**General and abdominal obesity**				
Age<60 / No abdominal obesity / BMI < 18.5	1.479 (1.323, 1.648)	2.418 (2.160-2.707)***	2.82 (1.22-6.50)*	2.689 (2.389-3.027)***
Age<60 / No abdominal obesity / 18.5 ≤ BMI < 27.5	1.194 (1.159, 1.230)	1	1	1
Age<60 / No abdominal obesity / 27.5 ≤ BMI < 30.0	1.023 (0.856, 1.213)	0.812 (0.683-0.965)*	0.71 (0.10-5.11)	0.791 (0.649-0.965)*
Age<60 / No abdominal obesity / 30.0 ≤ BMI < 35.0	1.294 (0.811, 1.959)	1.353 (0.890-2.058)	NA	0.923 (0.524-1.627)
Age<60 / No abdominal obesity / BMI ≥ 35.0	1.917 (0.049, 10.678)	1.505 (0.212-10.688)	NA	2.521 (0.355-17.901)***
Age<60 / Abdominal obesity / BMI < 18.5	2.542 (0.064, 14.161)	1.361 (0.192-9.666)	NA	6.434 (2.411-17.171)***
Age<60 / Abdominal obesity / 18.5 ≤ BMI < 27.5	2.138 (1.995, 2.288)	1.016 (0.943-1.094)	0.67 (0.37-1.21)	0.957 (0.877-1.046)
Age<60 / Abdominal obesity / 27.5 ≤ BMI < 30.0	1.625 (1.477, 1.785)	0.890 (0.807-0.982)*	1.00 (0.48-2.08)	0.854 (0.765-0.954)**
Age<60 / Abdominal obesity / 30.0 ≤ BMI < 35.0	1.718 (1.525, 1.927)	1.179 (1.046-1.329)**	0.34 (0.05-2.42)	1.011 (0.885-1.155)
Age<60 / Abdominalobesity / BMI ≥ 35.0	2.232 (1.672, 2.920)	2.107 (1.606-2.764)***	NA	1.798 (1.363-2.372)***
Age≥60 / No abdominal obesity / BMI < 18.5	56.269 (53.754, 58.870)	1.787 (1.670-1.913)***	1.02 (0.60-1.73)	1.929 (1.804-2.063)***
Age≥60 / No abdominal obesity / 18.5 ≤ BMI < 27.5	18.158 (17.861, 18.459)	0.936 (0.892-0.983)**	0.83 (0.57-1.21)	0.938 (0.891-0.988)*
Age≥60 / No abdominal obesity / 27.5 ≤ BMI < 30.0	11.020 (9.639, 12.544)	0.765 (0.667-0.877)***	0.75 (0.18-3.13)	0.729 (0.631-0.841)***
Age≥60 / No abdominal obesity / 30.0 ≤ BMI < 35.0	16.446 (11.174, 23.344)	0.964 (0.676-1.375)	NA	0.638 (0.406-1.003)
Age≥60 / No abdominal obesity / BMI ≥ 35.0	39.529 (12.835, 92.248)	1.618 (0.672-3.895)	NA	2.860 (1.189-6.881)*
Age≥60 / Abdominal obesity / BMI < 18.5	81.242 (53.985, 11.417)	1.986 (1.366-2.889)***	NA	1.841 (1.296-2.617)**
Age≥60 / Abdominal obesity / 18.5 ≤ BMI < 27.5	19.335 (18.792, 19.890)	0.874 (0.828-0.923)***	0.92 (0.61-1.38)	0.863 (0.815-0.914)***
Age≥60 / Abdominal obesity / 27.5 ≤ BMI < 30.0	14.681 (13.945, 15.446)	0.793 (0.741-0.849)***	0.61 (0.36-1.03)	0.738 (0.688-0.792)***
Age≥60 / Abdominal obesity / 30.0 ≤ BMI < 35.0	14.350 (13.223, 15.548)	0.803 (0.732-0.881)***	0.45 (0.23-0.88)*	0.763 (0.696-0.836)***
Age≥60 / Abdominalobesity / BMI ≥ 35.0	16.796 (12.617, 21.915)	1.100 (0.839-1.443)	0.31 (0.04-2.31)	1.194 (0.940-1.517)
**Cancer**				

None	4.418 (4.365, 4.472)	1	1	1
Cured or under-treatment patient	16.161 (15.626, 16.709)	1.382 (1.333-1.432)***	2.22 (1.67-2.93)***	1.499 (1.454-1.546)***
**Smoking packyear/Age ratio**				
Non-smoker	4.501 (4.434, 4.568)	1	1	1
0< to <1	5.238 (5.145, 5.332)	1.358 (1.318-1.399)***	0.87 (0.71-1.06)	1.308 (1.270-1.347)***
1+	11.151 (10.202, 12.166)	1.652 (1.509-1.809)***	1.31 (0.79-2.17)	1.749 (1.587-1.926)***
**Heavy alcohol drinking habit**				
Absence under the healthy condition	3.107 (3.053, 3.160)	1	1	
Presence under the healthy condition	3.592 (3.479, 3.708)	0.992 (0.955-1.031)	0.86 (0.72-0.94)**	0.896 (0.858-0.934)***
Absence/Presence under the unhealthy condition	13.609 (13.377, 13.843)	1.082 (1.054-1.111)***	1.06 (0.87-1.30)	0.958 (0.933-0.983)***
**Frequency of physical activity (times/week)**				
None	7.602 (7.462, 7.744)	1.323 (1.292-1.355)***	1.75 (0.95-2.70)	1.457 (1.423-1.492)***
1 or more	3.956 (3.899, 4.013)	1	1	1
**SBP and DBP (mmHg)**				
Normal	4.024 (3.970, 4.078)	1	1	1
HTN stage I (SBP 120 to <140 or DBP 90 to <100)	9.258 (9.046, 9.475)	1.052 (1.023-1.081)***	1.14 (0.50-2.56)	1.089 (1.060-1.120)***
HTN stage II (SBP 140+ or DBP 100+)	12.734 (12.048, 13.448)	1.219 (1.152-1.290)***	1.20 (1.00-1.43)	1.318 (1.243-1.397)***
**FBG level (mg/dL)**				
Normal (<100)	3.569 (3.513, 3.626)	1	1	1
Prediabetes (100 to <126)	6.424 (6.296, 6.554)	1.074 (1.046-1.102)***	1.12 (0.93-1.35)	1.026 (0.999-1.052)
Diabetes (126 to <200)	12.358 (11.975, 12.751)	1.350 (1.302-1.399)***	1.39 (1.07-1.79)	1.304 (1.260-1.350)***
Hyperglycemic crisis (200+)	14.800 (13.878, 15.767)	2.086 (1.953-2.229)***	1.67 (0.97-2.88)	2.118 (1.988-2.256)***
**Hgb level (g/dL)**				
Optimal (15+ for male; 14+ for female)	3.110 (3.036, 3.185)	1	1	1
Normal (13 to <15 for male; 12 to <14 for female)	4.559 (4.487, 4.632)	1.130 (1.097-1.164)***	1.32 (0.93-1.85)	1.288 (1.248-1.329)***
Anemia (<13 for male; <12 for female)	11.371 (11.122, 11.623)	1.760 (1.698-1.823)***	1.33 (0.97-1.85)	2.327 (2.245-2.412)***
**AST (SGOT) (U/L)**				
Optimal (≤ 40)	4.490 (4.436, 4.545)	1	1	1
Normal (40< to ≤50)	7.169 (6.816, 7.536)	1.145 (1.086-1.207)***	0.74 (0.44-1.26)	1.121 (1.064-1.182)***
High (>50)	11.704 (11.241, 12.180)	1.698 (1.621-1.779)***	1.72 (1.09-2.71)*	1.713 (1.638-1.792)***

**ɣ-GTP (U/L)**					
	Optimal (10< to ≤63 for male; 7< to ≤35 for female)	4.425 (4.368, 4.482)	1	1	1
	Normal (63< to ≤77 for male; 35< to ≤45 for female)	5.343 (5.083, 5.613)	1.174 (1.115-1.237)***	1.30 (0.87-1.95)	1.254 (1.189-1.322)***
	High (77< for male; 45< for female)	7.913 (7.695, 8.135)	1.583 (1.528-1.641)***	1.35 (1.03-1.78)*	1.777 (1.716-1.841)***
**eGFR (mL/min/1.73m2)**					
	Normal to mild (60+)	3.959 (3.908, 4.011)	1	1	1
	Mild to moderate damage (45 to <60)	13.866 (13.486, 14.254)	1.139 (1.104-1.176)***	1.20 (0.92-1.56)	1.182 (1.145-1.219)***
	Moderate to severe damage (<45)	17.476 (16.766, 18.209)	1.532 (1.464-1.602)***	2.43 (1.60-3.70)***	1.676 (1.641-1.849)***
**Urine protein in the Dipstick test (mg/dL)**					
	Negative / Trace / 1+ (<30)	4.550 (4.495, 4.604)	1	1	1
	2+ or more (30+)	16.901 (17.729, 20.129)	1.904 (1.784-2.033)***	2.11 (1.19-3.73)**	1.742 (1.641-1.849)***
**Cholesterol ratio**					
	Otherwise	4.548 (4.491, 4.606)	1	1	1
	At least one high§	6.373 (6.212, 6.538)	1.038 (1.008-1.068)*	1.10 (0.92-1.32)	1.148 (1.114-1.183)***

IR, incidence rate; AHR, adjusted hazard ratio; CI, confidence interval; BMI, body mass index; SBP, systolic blood pressure; DBP, diastolic blood pressure; Hgb, hemoglobin; AST, aspartate aminotransferase; γ-GTP, gamma glutamyl tranferase/transpeptidase; eGFR, estimated glomerular filtration rate; TC, total cholesterol; TG, triglycerides; HDL, high density lipoprotein; LDL, low density lipoprotein.

*, p<0.05; **, p<0.01; ***, p<0.001

§ At least one hight means one of (1) TC/HDL ratio ≥ 5.0, (2) LDL/HDL ratio ≥ 5.0, or (3) TG/HDL ratio >6.0.
